# Supplementary material for: Content-rich biological network constructed by mining PubMed abstracts
Source: BMC Bioinformatics. 2004 Oct 8;5:147. doi: 10.1186/1471-2105-5-147 (PMC528731; doi:10.1186/1471-2105-5-147)
Supplement: Additional File 2 — The original results of the above study (non-essential files are deleted to keep the file size under the limit set by BMC bioinformatics). [file 1471-2105-5-147-S2.bz2 › chilibotAdditionalFile2/dip05/320ID10576729E2441/html/ATPD_ATPG.html]

 


 **ATPD** and **ATPG** 
  
Found 7 abstracts in PubMed, retrieved 05.  
 

 What does Google say? 
 PDF only 
| .edu only 

---

**Interactive relationship** (e.g. stimulation, inhibition, etc)

**Non-interactive relationship** (e.g. studied together, co-existance, homology, etc.)

- In green plants, the genes  **atpG**  and  **atpD**  are located in the nucleus.  Ref: 1404401 J Mol Biol, 1992
- Relocation of atpIB to a position in the promoter distal region of the operon between  **atpG**  and  **atpD**  did not change the inactivation rate of atpB.  Ref: 8679701 Biochim Biophys Acta, 1996
- The C. thermoaceticum atp operon contained nine genes arranged in the order atpI i, atpB a, atpE c, atpF b, atpH delta, atpA alpha,  **atpG**  gamma,  **atpD**  beta, and atpC epsilon.  Ref: 9171425 J Bacteriol, 1997
- Eight genes atpI, atpB, atpE 1, atpE 2, atpE 3, atpF, atpH, and atpA upstream of and contiguous with the previously described genes  **atpG** ,  **atpD** , and atpC were cloned from chromosomal DNA of Acetobacterium woodii.  Ref: 10567365 J Biol Chem, 1999
